# Clinical outcomes in patients with advanced pelvic prolapse who underwent LeFort surgery or pessary placement- A prospective cohort study

**DOI:** 10.22088/cjim.13.2.405

**Published:** 2022

**Authors:** Elham Gholamian, Fedyeh Haghollahi, Samaneh Tarokh, Maryam Hajihashemy, Mamak Shariat

**Affiliations:** 1 Beheshti Hospital, Isfahan University of Medical Sciences, Isfahan, Iran; 2 Vali-E-Asr Reproductive Health Research Center, Family Health Research Institute, Tehran University of Medical Sciences Tehran, Iran; 3 Isfahan University of Medical Sciences, Isfahan, Iran; 4 Maternal, Fetal, and Neonatal Research Center, Family Health Research Institute, Tehran University of Medical Sciences. Tehran, Iran

**Keywords:** Modified LeFort colpocleisis, Pelvic floor reconstructive surgery, Pelvic organ prolapse

## Abstract

**Background:**

The aim of the present study was to compare the six-month results in terms of prolapse symptoms in postmenopausal patients with advanced pelvic prolapse (POP) who underwent LeFort colpocleisis surgery or with pessary placement.

**Methods:**

In this prospective cohort study, 110 older women were enrolled from April 2016 to January 2018. The women were diagnosed with stage III or higher genital prolapse according to the POP quantification (POP-Q) system. The patients were divided into two groups: surgical (LeFort colpocleisis surgery; n=55) and non-surgical (pessary placement, n=55). The study population underwent LeFort colpocleisis surgery or pessary placement in two university clinics (Beheshti or Alzahra Hospitals). All patients completed the Pelvic Floor Distress Inventory Questionnaire-20 (PFDI-20). The main short-term outcome measurement (six months) was the manifestation of a pelvic prolapse in the groups.

**Results:**

The patients had a mean age of 68.98±8.79 years in the non-surgical group and 64.76±7.04 years in the surgical group. The analytic results showed a significant difference between the two groups (P=0.006). After treatment, the prolapse symptoms improved in both groups (p<0.001). However, the total PFDI-20 score did not show any significant differences at the end of the six-month follow- up (P=0.19).

**Conclusion:**

Both strategies (pessary placement or LeFort colpocleisis surgery) provide a short-term option for the treatment of older women with stage III or higher POP. The findings of this study could assist with treatment options and allow better guidance for older patients with symptomatic POP in the treatment decision process.

Pelvic organ prolapse (POP) includes prolapse in the uterus, bladder, urethra, or rectum due to a defect in the pelvic support system, and is a common abnormality among older women (1). Although the prevalence of POP is unknown because numerous women do not seek medical care (2), it is more frequent with increasing age. In 10% of women aged 20 to 39 years and in 60% of those over 60 years, POP has been reported. The predisposing factors for this complication include heredity, female sex, pregnancy, childbirth, hysterectomy, myopathy, and neuropathy (3). A negative impact on quality of life domains was reported in symptomatic urogenital prolapse (2). Due to increased life expectancy and improved quality of life in women, POP has become a significant issue and its incidence is predictable to double in the next 25 years (1). There are two types of treatment for POP, the surgical and non-surgical.

Pelvic floor exercises, physiotherapy, and the use of supportive devices such as vaginal pessaries comprise the non-surgical treatments that are effective in improving pelvic floor dysfunction (4-7). Reconstructive and obliterative surgeries such as sacrospinous ligament suspension or sacrocolpopexy or Lefort surgery is recommended in general for women whose prolapse symptoms have not improved with noninvasive treatments like a pessary. Colpocleisis is less invasive than reconstructive surgery (8).

The woman's general health, the degree of prolapse, need for sexual intercourse, reproductive function, and the presence or absence of urinary symptoms are important factors to consider when choosing the appropriate treatment. In general, older women or those who are medically unfit and have no desire for sexual intercourse control their prolapse symptoms with a pessary. However, these are not universally successful and may not be acceptable to some women (8). 

 Although surgery’s primary aim is to repair the anatomy and develop the quality of life, there are few studies about the results of this treatment on the bladder, intestinal, and sexual functioning (9). In addition, there are significant implications for the costs of prolapse surgery, especially when the surgery has a failure rate of up to 30% (10). Urinary tract infections, post-treatment infections, sexual dysfunction, bleeding and pain, hospitalization, and the impact on the quality of life are the complications of treatment in two surgical or non-surgical procedures (11-19). Similar improvements have been reported in urinary and bowel symptoms, sexual function, and quality of life in both POP surgery and pessary placement (20, 21). "Colpocleisis involves the closure of the vagina and seems to have fallen out of fashion. In addition, recent reports have questioned the use of LeFort’s procedure and feel that it has no place in modern gynecological practice" (8,22, 23). However, advantages of this surgery include a lack of injury to the nearby organs, vessels, or nerves (24); rapid surgery; short recovery time; and use of local anesthesia, if necessary. Because of the high prevalence of POP among the middle and older aged women, the importance of women's health, and the Ministry of Health and Medical Education attention to this issue, this survey aimed to compare the clinical short-term outcomes in the LeFort colpocleisis surgery and pessary placement in patients with advanced POP.

## Methods

The Ethics Committee of Isfahan University of Medical Sciences, Isfahan, Iran and the Deputy of Research at Isfahan University of Medical Sciences, Isfahan, Iran (IR.MUI.MED.REC1397.048) approved this prospective cohort study. This study was registered at the Iranian Registry of Clinical Trials (No: IRCT20160521027998N6). The patients signed an informed consent to partake in this study.

A total of 110 postmenopausal patients with symptomatic advanced POP (stage III and higher) based on the POP quantification (POP-Q) system enrolled in this study. The study patients were divided into two groups, surgical (LeFort colpocleisis surgery; n=55) or non-surgical (pessary placement; n=55). The procedures were performed in two university clinics of Isfahan (Beheshti or Alzahra Hospitals) from April, 2016 to January, 2018.

 The exclusion criteria were patients with genital infections, history of bleeding, pelvic malignancy, history of pelvic floor surgery for prolapse and their recurrence or pessary insertion, and those who used narcotic drugs. The study patients were informed that participation included an interview and pelvic examination**.**

All of the pessaries were made of silicone (Gelhorn, Barcelona, Spain) and were placed by a single gynecologist. In the LeFort surgery, the vaginal mucus is bonded to the anterior-posterior plane, which results in a type of blockage of the vagina that prevents the removal of prolapsed organs (25). The lifetime risk of POP repair is estimated at 11%, and failure in the initial repair and re-functioning is 29% (26).

The Pelvic Floor Distress Inventory Questionnaire-20 (PFDI-20) contains 20 items and is divided into three subscales: Pelvic Organ Prolapse Distress Inventory (POPDI-6), the Colorectal-Anal Distress Inventory (CRADI-8), and the Urinary Distress Inventory (UDI-6). The first part of the POPDI-6 questionnaire examines the six manifestations of prolapse and includes the heaviness feeling in the pelvis, pain under the abdomen, vaginal protrusion; need to strain to pass urine and feces, feeling of incomplete discharge of urine, and vaginal pressure to urinate. 

The second part of the questionnaire (CRADI-8) evaluates the eight manifestations of bowel dysfunction, and includes excessive straining for defecation, lack of full discharge of feces, fecal incontinence with soft consistency, intestinal gas incontinence, painful discharge of feces, urgency in fecal defecation, and rectal prolapse. The third part of this questionnaire (UDI-6) consists of six urinary incontinence manifestations and includes repeated urination; urinary leakage associated with urgency; urinary leakage by sneezing, coughing or laughing; a few drops of urinary leakage; lack of complete discharge of urine; and pain or discomfort in the genital area and under the abdomen (27). The study participants were asked if they experienced each symptom and their responses were based on a Likert scale of 1–4, as follows: 1 (not at all), 2 (somewhat), 3 (moderate), and 4 (quite a bit). The total score for each subscale ranged from 1 to 80 (28). The higher score indicated a high severity of prolapse.

All patients completed the validated version of the PFDI-20 before and six months after the intervention. The comparison of changes in the PFDI-20 scores was the main outcome. The data gathering tool was a demographic information questionnaire that included age, body mass index (BMI), education level, and number of deliveries. Both the data gathering tool and another questionnaire were completed by interviewing the research participants. The degree of satisfaction was recorded based on the patients’ responses in the two groups and a score of 1–4 was given based on the Likert scale. 

**Statistical analysis: **We calculated the sample size according to the comparison of mean, which was reported by Anantawat et al. (13). We chose 55 patients per group, which indicated a 95% significance level, 80% power, and 10% loss to follow-up estimation.

Statistical analyses were performed using SPSS software (Version 20, SPSS, Inc., IL, USA). Data are expressed as mean±standard deviation and numbers with percentages. The paired t- test was used to compare between continuous scores before and after the treatments. We used the independent t-test for comparison between continuous variables between the two groups. Categorical variables were compared using the chi-square tests. A p-value <0.05 was considered to be statistically significant.

## Results

**Figure 1:**
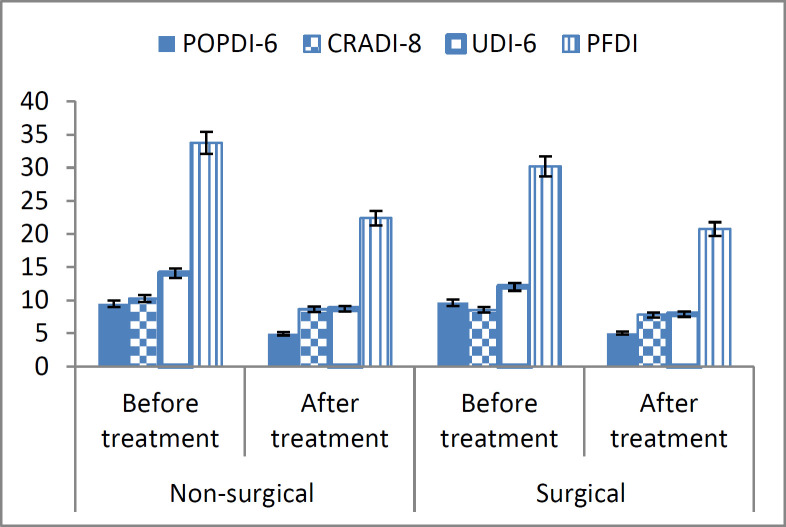
Comparison of clinical outcomes in each group (surgical and non-surgical) pre and post-treatments

POPDI-6: Pelvic Organ Prolapse Distress Inventory; CRADI-8: Colorectal-Anal Distress Inventory; UDI-6: Urinary Distress Inventory; PFDI-20: Pelvic Floor Distress Inventory Questionnaire-20.

At first, a total of 120 patients with 2 surgical (n=60) and non-surgical (n=60) groups were included in the study. Of these, 10 refused to participate due to unwillingness, including 5 in the surgical group and another in the non-surgical group. (Figure 1). Patients were assessed before and in the six-month follow-up after the final treatment. All 110 participants finalized the study and their data were considered in the analysis. The mean age was 68.98±8.79 years in the non-surgical group and 64.76±7.04 years in the surgical group. 

The t-test results showed a significant difference between the two groups (P=0.006). Table 1 summarizes the characteristics of the enrolled women. Statistically, demographic characteristics did not differ between the two groups (table 1). However, prior to treatment, significant differences in the CRADI and UDI domains were reported between the two groups. The non-surgical group reported a higher severity of prolapse symptoms in the CRADI and UDI domains (p<0.05). After treatment, the CRADI domain of the PFDI-20 questionnaire had a borderline significant difference between the two groups (P=0.05), but there were no statistically significant differences in terms of UDI (P=0.21) and POPDI (P=0.81) between the groups. In other words, the effects of the pessary placement or LeFort colpocleisis surgery on these domains was the same (p>0.05) (table 2). At the end of treatment, the total satisfaction score was not different between the non-surgical and surgical (P=0.64) groups. A total of 49 (89.1%) patients in the non-surgical and 51 (92.7%) in the surgical group stated they were satisfied with the reduction in POP symptoms. The dissatisfaction stated by 6 (11%) patients in the pessary group was attributed to the need for frequent referrals to check the device and the problem with sexual intercourse. Dissatisfaction in 4 (7.3%) patients in the surgical group was due to perineal pain. After the end of treatment, the total PFDI-20 score did not show any differences between the two groups (P=0.19). The PDFI-20 scores showed improvement after treatment in both groups. Therefore, the POP symptoms improved in the non-surgical and surgical groups (table 3, figure 2). There were no serious adverse effects observed in either group. The patients were treated successfully and no complications were found during the six-month follow-up.

**Table 1:** Baseline characteristics in the two study groups

*P-value	**Surgical group** **(LeFort colpocleisis surgery)** **n=**55	**Non-surgical group (pessary placement)** **n=55**	**Variable **
0.006	64.76±7.04	68.98±8.79	Age (years)
0.33	28.32±3.69	27.66±3.30	BMI (kg/m^2^)
0.39	3.91±1.46	4.15±1.42	Parity
0.53	48 (87.3) 7 (12.7)	51 (92.7) 4 (7.3)	Job; n (%) Housewife Employee
0.57	15 (27.3) 26 (47.3) 14 (25.5)	19 (34.5) 26 (47.3) 10 (18.2)	Education; n (%) Illiterate ≤Diploma Graduate
1	11 (20) 44 (80)	10 (18.2) 45 (81.8)	Hysterectomy; n (%) Yes No
0.17	9 (16.4) 46 (83.6)	16 (29.1) 39 (70.9)	Grade of prolapse; n (%) 3 4

BMI: Body mass index. Data are expressed as mean±standard deviation and number with percentages in parentheses.

*P-values refer to the t-test and chi-square test when necessary.

**Table 2:** Comparison of clinical outcomes before and after treatment between the surgical and non-surgical groups

*P-value	**Surgical group** **(LeFort colpocleisis surgery)** **n=**55	**Non-surgical group (pessary placement)** **n=55**	**Variable**
**Before ** **t** **reatment**			
0.80	9.65±4.73	9.45±3.44	POPDI-6
0.007	8.55±2.87	10.24±3.56	CRADI-8
0.02	12.02±5.29	14.07±3.74	UDI-6
0.08	30.22±11.40	33.76±9.14	PFDI-20
**After ** **t** **reatment**			
0.81	5.04±2.28	4.95±1.72	POPDI-6
0.05	7.80±1.79	8.64±2.55	CRADI-8
0.21	7.91±3.40	8.69±3.11	UDI-6
0.19	20.75±6.37	22.38±6.74	PFDI-20
0.64	4.27±0.76	4.35±0.87	Satisfaction
0.38	3 (5.5%) 1 (1.8%) 29 (52.7%) 22 (40%)	4 (4.3%) 2 (3.6%) 20 (36.4%) 29 (52.7%)	**Satisfaction ** **a** **fter ** **t** **reatment** Unsatisfied No change Satisfied Very satisfied

Data are expressed as mean±standard deviation and number with percentages in parentheses.*P-values refer to the t-test and chi-square test when necessary. POPDI-6: Pelvic Organ Prolapse Distress Inventory; CRADI-8: Colorectal-Anal Distress Inventory; UDI-6: Urinary Distress Inventory: PFDI-20: Pelvic Floor Distress Inventory Questionnaire-20

**Table 3:** Comparison of clinical outcomes pre- and post-treatment within each group

*P-value	**After treatment**	**Before treatment **	**Group**
**Surgical group (LeFort colpocleisis)**			
<0.001	4.95±1.72	9.45±3.44	POPDI-6
0.001	8.64±2.55	10.24±3.56	CRADI-8
<0.001	8.69±3.11	14.07±3.74	UDI-6
<0.001	22.38±6.74	33.76±9.14	PFDI-20
**Non-surgical group (Pessary)**			
<0.001	5.04±2.28	9.65±4.73	POPDI-6
0.003	7.80±1.79	8.55±2.87	CRADI-8
<0.001	7.91±3.40	12.02±5.29	UDI-6
<0.001	20.75±6.37	30.22±11.40	PFDI-20

* P-value refers to the paired t-test.

POPDI-6: Pelvic Organ Prolapse Distress Inventory; CRADI-8: Colorectal-Anal Distress Inventory; UDI-6: Urinary Distress Inventory; PFDI-20: Pelvic Floor Distress Inventory Questionnaire-20

## Discussion

In older women, both pessary placement and LeFort surgery appeared to be effective for the cure of stage III or higher genital prolapse. Both groups reported reductions in scores from the three domains (UDI, CRADI, and POPDI), which indicated the effectiveness of both treatments.

Lotte et al. conducted a prospective cohort study in women with symptomatic stage II or greater POP.113 women were treated according to their preference with either pessary placement or prolapse surgery. The prolapse domain of the UDI questionnaire was completet in each patient 12 months after the treatments. The results showed that the prolapse symptoms in those who were treated with a pessary were less severe than the surgery group (18). 

Lamers et al., in a review article, found that one year outcomes in terms of prolapse symptoms appeared to be similar between pessary placement and surgery (29). Miceli and Dueñas-Diez conducted a prospective observational study with 171 women who had symptomatic advanced POP. The patients were treated according to their preference (surgery or vaginal ring pessary) and were followed for a minimum of 18 months. The study results showed that the efficacy (recurrent prolapse) was similar in both groups. The pessary group had a success rate of 84.4% compared with 89.6% in the surgery group (30).

 The results reported by the Lamers et al. and Miceli and Dueñas-Diez studies were consistent with the present study (29-31). The study of Radnia, et al. on women ranging from 34 to 89 years in 6 months follow-up,and based on PDFI-20 questionnaire showed that in many patients, especially the older ones; pessaries improve symptoms of prolapse and can be substituted for surgery (32). The reason for the differences in the results of these two studies is the use of different pelvic prolapse assessment questionnaires,the age of women, and the follow-up periods. In addition, there are extensive variations in the definitions of success in pelvic prolapse surgery (27,33). In the present study, consistent with Radnia’s study (32) ,we used validated questionnaires to assess the objective manifestation outcomes. Manifestations of the pelvic prolapse symptoms indicate the patient's response in elderly patients. 

Limitations of the current study included the small sample size, non-randomized trial, short-term outcome, and the limited availability of published studies that pertained to this issue. We did not assess the health quality of life or other complications in this research. These measures and possible confounding factors should be considered in future studies. We recommend that a randomized controlled trial, which compares the pessary to POP surgical treatments is recommended to determine the therapeutic position and the management of POP.
